# A comparison of the existential and medical models of addiction

**DOI:** 10.1192/j.eurpsy.2021.1509

**Published:** 2021-08-13

**Authors:** G. Grech

**Affiliations:** Psychiatry, Mount Carmel Hospital, Attard, Malta

**Keywords:** medical model, choice and responsibility, existential model, Addiction

## Abstract

**Introduction:**

After developing an existential model of addiction, it became evident that there are major differences between the existential and medical models of addiction.

**Objectives:**

This research aims to investigate the boundary and overlap between the existential and medical models of addiction.

**Methods:**

The existential model was compared and contrasted with a narrative literature review of the medical model of addiction.

**Results:**

Through the existential definition being-with-drug, addiction is conceptualised in terms of a relationship with the drug and the impact on one’s sense of self. The medical model focuses on diagnostic criteria, genetic and environmental risk and protective factors, and an underlying neurobiological explanation. In contrast to the prevalent disease model, the existential view maintains that drug addiction is a coping mechanism used to mitigate existential and neurotic anxiety which results from facing or avoiding the existential givens. Phenomenological research supporting existential psychotherapy in addiction is contrasted with the quantitative medical research which forms the basis for current addiction guidelines. A comparison of both models is presented focusing on the issues of coping, choice, responsibility, mandatory treatment, medication, psychotherapy and the therapeutic relationship. The biopsychosocial model is compared to van Deurzen’s modes of existence, which provides the basis for existential psychotherapeutic interventions. Furthermore, existential literature was examined to determine whether an individual can authentically choose to live addicted.

**Conclusions:**

Both models fall short of giving a holistic view of addiction. A combination of models is necessary to address the diversity of issues patients present with.

